# Follicular Fluid Metabolomics: Tool for Predicting IVF Outcomes of Different Infertility Causes

**DOI:** 10.1007/s43032-024-01664-y

**Published:** 2024-08-01

**Authors:** Yijing Zhang, Chenyan He, Yuedong He, Zhongyi Zhu

**Affiliations:** 1 Department of Obstetrics and Gynecology, West China Second University Hospital, Sichuan University, Chengdu, China; 2 Key Laboratory of Birth Defects and Related Diseases of Women and Children (Sichuan University), Ministry of Education, Chengdu, China; 3 Sichuan Normal University, Chengdu, Sichuan China

**Keywords:** Follicular fluid, Metabolomics, In vitro fertilization, Oocyte quality, Embryo viability

## Abstract

Infertility affects approximately 15% of couples at child-bearing ages and assisted reproductive technologies (ART), especially in vitro fertilization and embryo transfer (IVF-ET), provided infertile patients with an effective solution. The current paradox is that multiple embryo transfer that may leads to severe obstetric and perinatal complications seems to be the most valid measure to secure high success rate in the majority of clinic centers. Therefore, to avoid multiple transfer of embryos, it is urgent to explore biomarkers for IVF prognosis to select high-quality oocytes and embryos. Follicular fluid (FF), a typical biofluid constituted of the plasma effusion and granulosa-cell secretion, provides essential intracellular substances for oocytes maturation and its variation in composition reflects oocyte developmental competence and embryo viability. With the advances in metabolomics methodology, metabolomics, as an accurate and sensitive analyzing method, has been utilized to explore predictors in FF for ART success. Although FF metabolomics has provided a great possibility for screening markers with diagnostic and predictive value, its effectiveness is still doubted by some researchers. This may be resulted from the ignorance of the impact of sterility causes on the FF metabolomic profiles and thus its predictive ability might not be rightly illustrated. Therefore, in this review, we categorically demonstrate the study of FF metabolomics according to specific infertility causes, expecting to reveal the predicting value of metabolomics for IVF outcomes.

## Introduction

Infertility is diagnosed when a couple failed to achieve pregnancy after 12 months or more of regular sexual intercourse without contraception. Under the circumstances of increasing severe pollution and pressure worldwide, infertility affects approximately 15% of couples at child-bearing ages reported by the National Institute of Health and Human Development (NIH) and has become a prevalent global health problem as World Health Organization suggested (WHO) [1, 2].

As the incidence keeps climbing for recent years, assisted reproductive technologies (ART), especially in vitro fertilization and embryo transfer (IVF-ET), has provided infertile spouses with an alternative and optimal therapy to solve infertility, which dramatically increased the pregnancy success rate to 25–60% varying among different centers and the types of embryo transferred [3]. However, the high success rates are partially depended on transfer two or more than two embryos simultaneously, leading to a series of obstetrics and perinatal complications caused by multiple gestations, including abortion, preterm birth, low birth weight and small size for gestational age [4]. To reduce multiple births, the elective single-embryo transfer (eSET) was firstly introduced to IVF in 1999 and is prevalently encouraged nowadays [5]. As eSET experimentations demonstrated, about one-third overall IVF live-births were absent owing to decreased twin births and pregnancy rates [6]. Therefore, persistent effort has been made to explore and establish models to predict the IVF prognosis including oocyte quality, embryo viability, implantation rate and live-birth rate in a cycle to raise the success rate, alleviating patients’ physical, psychological and financial burden [7].

Follicular fluid (FF), mainly constituted of the effusion of blood plasma in the capillaries of the ovarian cortex along with secretion of theca cells and granulosa cells in the follicle, provides relatively independent in vivo microenvironment for oocytes development. FF contains a series of components essential for oocyte maturation primarily including protein, steroid hormones, cytokines, superoxide, antioxidants, growth factors and various metabolites [8–10], serving as an important intermediary for the communication between oocytes and their surrounding cells within follicles. On the one hand, FF provides essential intracellular substances such as amino acids, lipids and nucleotides for oogenesis and ovum maturation which consequently influences gametes fertilization, and early embryo development [11, 12]. On the other hand, the accumulation of harmful substances in the follicular fluid will also hinder ovum maturation by aberration of ovum microtubule tissue and abnormal arrangement of chromosome. Due to the finding that variation in FF composition reflects oocyte developmental competence and embryo viability, an increasing number of investigations into the metabolic profile of FF have been carried out to provide potential biomarkers for oocyte quality, embryo viability and even clinical pregnancy, seeking for supplemental assessment method for ART [13]. As a by-product of follicular aspiration during in vitro fertilization procedures, FF may be an ideal study object considering its potent sample source and convenient acquisition and to a certain extent, FF research can be regarded as non-invasive, since obtaining FF cause no extra injury to patients.

**Fig. 1:**
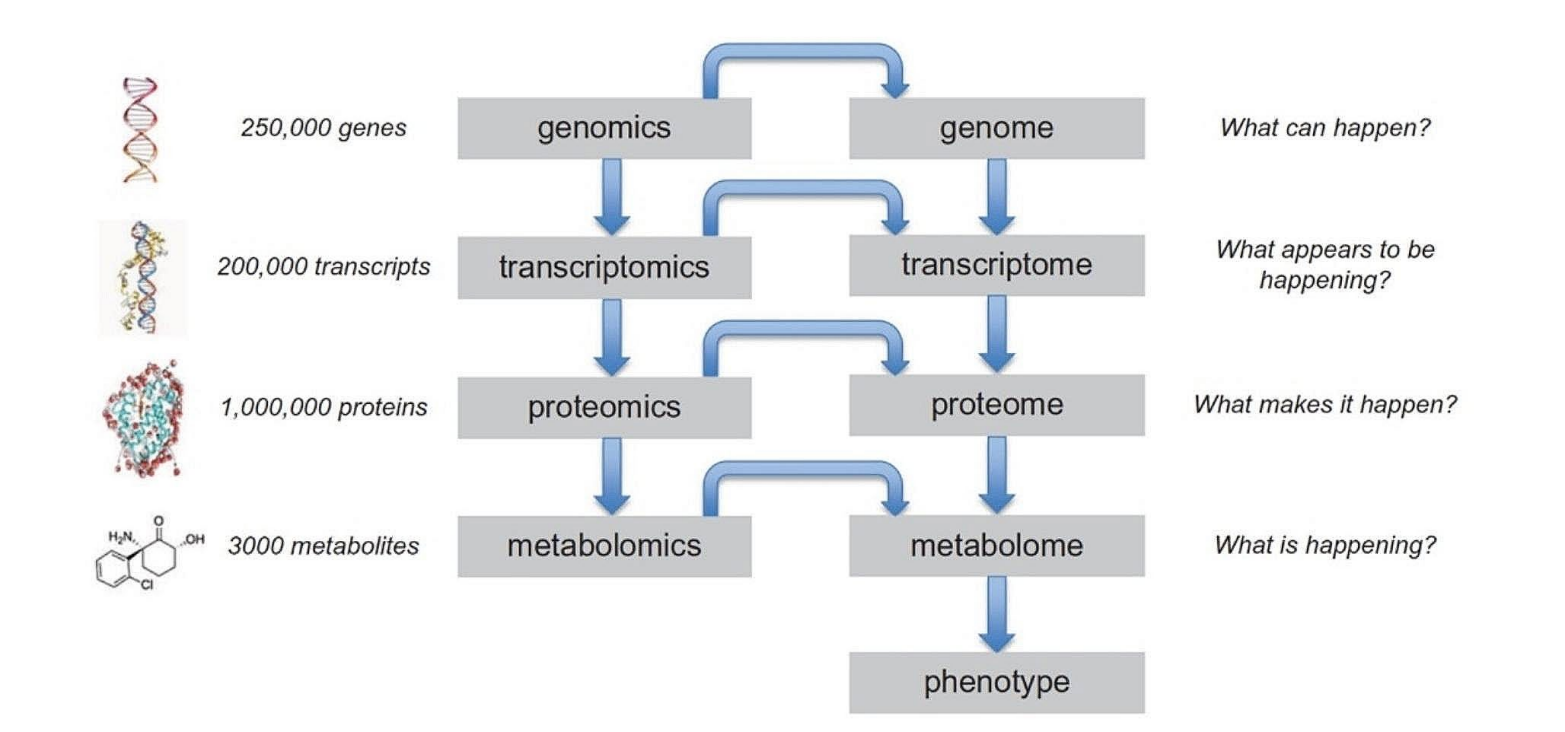
The ‘omics’ cascade describes the flow of biological information in an organism [17]

Metabolomics, encompassing an integral set of metabolites of a specific biological sample, is found to be capable of determining the link between metabolic end-products and physiological and pathological alteration through quantitative analysis. It is able to dynamically response to even the slightest chemical signals [14]. Metabolomics not only comprises the final downstream products of gene expression, offering information about genotype–phenotype-environment relationships, but also have an advantage over other omics since the human genome consists of over 0.25 million genes which subsequently encode for over 1 million proteins, while only about 3 thousands metabolites comprise the whole human metabolome (Fig. 1), allowing faster analyses of metabolomics in comparison to genomic and proteomic [15–17]. Additionally, as an analytical method for the systematic detection of metabolic profiles, metabolomics has the advantage of high accuracy, high sensitivity and large throughput in component analysis of several kinds of bio-fluids. Common metabolomics techniques include liquid chromatography-tandem mass spectrometry (LC-MS/MS), gas chromatography-mass spectrometry (GC-MS/MS), capillary electrophoresis-mass spectrometry (CE-MS) and nuclear magnetic resonance spectroscopy (NMR) [18, 19].

**Fig. 2:**
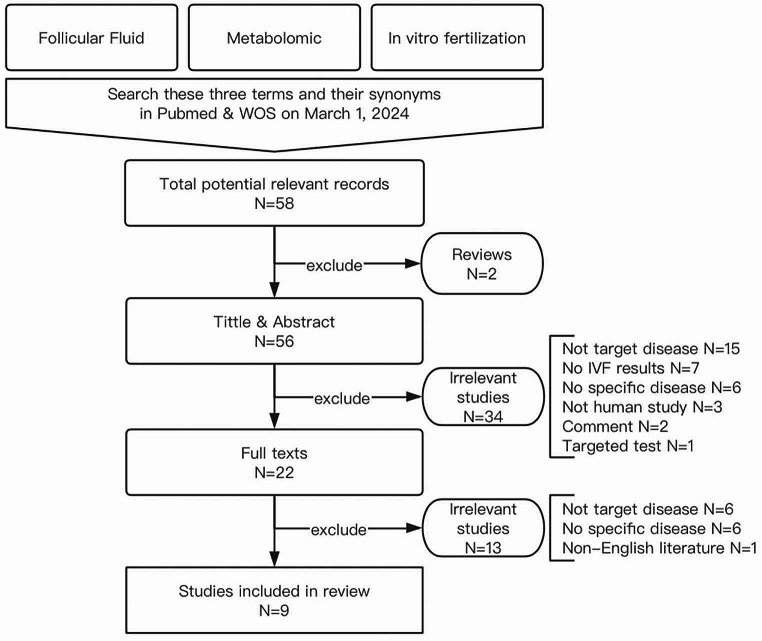
The search strategy and data selection flow diagram for the review

Since the analysis of human-embryos secretome by metabolic foot printing was firstly reported in 2007, scientists have attempted to use metabolomics to determine potential biomarkers in FF on purpose of improving IVF outcomes [20, 21]. The composition of follicular fluid was found to be different from that of plasma with characteristic of lipid localization. Furthermore, the metabolic profiling of follicular fluid was found to be correlated with the developmental competence of the human oocyte, suggesting that FF metabolomics may be a promising technique in gamete and embryo selection [21]. Although the study of FF metabolomics has unveiled considerable information of the mechanism of infertility and provided a great possibility for screening markers with diagnostic and predictive value, its effectiveness and practicability has drawn some skepticism [17]. One of the reasons may be that current reviews on metabolomics searching for markers of IVF outcome are mostly stated according to classification of different biofluids and tend to ignore the impact of sterility causes on the FF metabolomics whilst varied diseases have been clarified to have differed metabolic profiles [22, 23]. Therefore, in this paper, we categorically discuss the predicting value of metabolomics for IVF outcomes in terms of different infertility-related diseases (Table 1; Fig. 2).

**Table 1:** Metabolomic indicators in FF for IVF outcomes

Disease	Author	Sample and method	Differential metabolites	Correlation with IVF outcomes
PCOS	Ding Y et al. (2022)	Pilot study. 25 women with PCOS and 12 women without PCOS undergoing IVF. LC-MS/MS	LPG,18:0.	Positively correlated with high-quality embryo rate.
			Ceramide, FFA (Cer,36:1;2, Cer,38:1;2, Cer,38:2;2, Cer,40:0;2 and FFA C12:0).	Negatively associated with high-quality embryo rate.
	Feng Y et al. (2022)	Case control study. 64 non-obese women (32 with PCOS and 32 age- and BMI-matched controls) undergoing IVF GC-MS	PGE2 and PGJ2.	Negatively correlated with high-quality embryo rate.
	Guan S Y et al. (2022)	Case control study. 30 women with PCOS and 30 women with the fallopian tubal issues undergoing IVF. UHPLC-QE-MS	LysoPE (16:0/0:0), DG (18:2(9Z,12Z)/15:0/0:0), Linoleyl carnitine and Androsterone sulfate.	Predictors of abortion rate.
			DG (15:0/18:3(6Z,9Z,12Z)/0:0) and LysoPA (18:1(9Z)/0:0).	Predictor of the live birth rate and delivery rate.
			LysoPA (18:1(9Z)/0:0).	Predictor of pregnancy rate.
Endometriosis	Marianna S et al. (2017)	Pilot study. 16 patients with endometriosis. 1 H-NMR	Glucose.	Positively correlated with oocyte quality.
			Insulin and lipid.	Correlated with reduced oocyte quality.
	Dabaja M Z et al. (2022)	Case control study. 7 patients with endometriosis (3 failed to get pregnant and 4 got pregnant after ICSI). ESI-HRMS	PA 35:6 and PA 37:7	Biomarkers for pregnancy.
DOR	Liang C et al. (2021)	Case control study. 20 patients with DOR and 20 patients with tubal factors after micro-stimulation strategy. UHPLC-MS-MS	20-HDoHE, ± 5-iso PGF2α-VI, 12 S-HHTrE, 8 S,15 S-DiHETE,1a,1b-dihomo PGE2, 20-COOH-AA, PGA2, PGE1 and PGF2α.	Positively correlated with the number of oocytes retrieved, MII oocytes and fertilization.
			20-COOH-AA	Positively correlated with the number of high-quality embryos.
Uterine cavity abnormalities	Dabaja M Z et al. (2022)	Case control study. Patients with endometriosis ( 3 pregnant and 4 non-pregnant after IVF). ESI-HRMS	GlcCerC (d18:0/20:0), Phosphatidylethanolamine, PC (16:0/16:0), PG (O-18:0/16:1(9Z)), PE (O-18:0/22:2(13Z,16Z)) and GluCer d18:1/24:0	Biomarkers for pregnancy.
Fallopian impairment	Montani D A et al. (2019)	Case control study. Patients with tubal factor and/or mild male factor (28 pregnant and 34 non-pregnant) after ICSI. MALDI-TOF mass spectrometry	Phosphatidic acid, triacylglycerol and phosphatidylglycerol.	Hyper-represented in the pregnant group, assisting in building pregnancy prediction model.
	Castiglione Morelli M A et al. (2020)	Pilot study. 23 infertile patients with tubal factor undergoing ICSI. 1 H NMR	Lipid and cholesterol.	Positively correlated with the number of total oocytes.
			Glutamate.	Positively correlated with the number of MII oocytes.
	Guan S Y et al. (2022)	Case control study. 30 women with the fallopian tubal issues. UHPLC-QE-MS	LysoPE (16:0/0:0) and DG (18:2(9Z,12Z)/15:0/0:0).	Predictors of pregnancy rate, delivery rate and live birth rate.
	Liu A et al. (2023)	Case control study. 35 infertile patients with tubal factor ICSI. UPLC-MS/MS	Tryptophan.	Positively correlated with the available embryo rate.

PCOS: polycystic ovary syndrome; DOR: diminished ovarian reserve; LC-MS/MS: liquid chromatography-tandem mass spectrometry; GC-MS: gas/liquid chromatography-mass spectrometry; UHPLC-QE-MS: ultra-high-performance liquid chromatography-mass spectrometry; MALDI-TOF: matrix-assisted laser desorption/ionization-time of flight; NMR: nuclear magnetic resonance spectroscopy; UPLC-Q-TOF: ultra-performance liquid chromatography-quadrupole-time of flight-mass; ESI-HRMS: high-resolution electrospray ionization mass spectrometry; ICSI: intracytoplasmic sperm injection LPG: lysophosphatidylglycerol; FFA: free fatty acid; PGE: prostaglandin E; PGJ: prostaglandin J; LysoPE: lysophosphatidylserine; DG: D-Glutamine; LysoPA: lysophosphatidic acid; PA: Phosphatidic acids; PGF:; AA: amino acid; PC: phosphatidylcholines; PG: phosphatidylglycerol; PE: phosphatidylethanolamines

## FF Metabolomics in PCOS

Polycystic ovary syndrome (PCOS) affects 10–13% of women [24] in reproduction, metabolism, and psychological condition across the lifespan. The cause includes genetic and epigenetic susceptibility, hypothalamic and ovarian dysfunction, excess androgen exposure, insulin resistance, and adiposity-related mechanisms [25]. It was recommended by International Evidence-based Guideline that PCOS should be diagnosed with the 2018 International Evidence-based Guideline criteria promoted from 2003 Rotterdam criteria. Two of the following is required: (1) clinical/biochemical hyperandrogenism; (2) ovulatory dysfunction; (3) polycystic ovaries on ultrasound which can be alternatively substituted by anti-Müllerian hormone (AMH). Importantly, other etiologies should be excluded. Besides, where irregular menstrual cycles and hyperandrogenism are present, ultrasound or AMH are not required for diagnosis [26]. The clinical manifestations of PCOS include oligomenorrhea or amenorrhea, hirsutism, and frequently infertility [27].

According to the World Health Organization, ovulatory disorders account for approximately 25% of infertility diagnoses [28]. PCOS, as the most common cause of anovulation, attribute to 70% of ovulation disturbance [27]. Statistically, the global prevalent cases of infertility among women of reproductive age due to PCOS reached up to 12.13 million in 2019 and the number keeps increasing [29]. Although some PCOS patients could conceive babies undergoing assisted reproductive techniques (ART), the reproductive outcomes of these women tend to be poorer when compared to those with other causes of infertility [30, 31]. It is of concern that although PCOS patients could produce morphologically normal metaphase II oocytes, the developmental competence of these oocytes and the subsequent embryos could be impaired and lead to failure in IVF procedures. This may be caused by dysfunctions in the metabolism of glycerolipid, glycerophospholipid, sphingolipid, and glycosphingolipid biosynthesis in follicles which affects the 2 pronuclei (PN) fertilization rate during IVF procedure [32]. Therefore, researches have been focused on exploring metabolomic biomarkers for screening eggs and embryos of high quality to improve the IVF outcomes for PCOS infertile women.

As a metabolic-disorders related syndrome, PCOS alters FF metabolic profiles. Women with PCOS has significantly different metabolomic features from those without PCOS in FF. A pilot study by nuclear magnetic resonance (NMR) spectroscopy revealed significant decreases in the levels of acetate, β-hydroxybutyrate, leucine and threonine, as well as a reduction of lactate and significant increases in the levels of glucose, creatine and glycerol in PCOS patients [33]. Metabolomic analysis by ultra-performance liquid chromatography mass spectrometry (UPLC-MS) also uncovered that 14 biomarkers including fatty acids, diacylglycerol, triacylglycerol, ceramide, ceramide-phosphate, phosphatidylcholine and sphingomyelin were of decreased abundance in the PCOS group when compared to the control group composed of patients who were being treated mainly for tubal factor or mild male factors of infertility [31]. Furthermore, ceramide, free fatty acids and lipid subclasses provide not only diagnostic biomarkers of PCOS itself, but also candidate biomarkers to screen out oocyte of high quality for improving IVF outcomes [34]. Follicular fluid levels of PGE2 and PGJ2 were negatively correlated with high-quality embryo rate in PCOS patients as a result of oocyte competence impair [35]. A total of 11 kinds of biomarkers were verified to be associated with clinical outcomes including pregnancy rate, delivery rate and live birth rate of ART companied by a series of metabolic biomarkers such as androsterone sulfate, Glycerophosphocholine, and elaidic carnitine robustly predicting the abortion rate of the PCOS group [36]. Metabolomic study allows the comparison of FF from individuals with or without PCOS and therefore offers a whole set of promising biomarkers to enhance IVF outcomes for PCOS patients. However, its technical strength and clinical practicability need further researches of lager quantity and higher-level evidence.

## FF Metabolomics in Endometriosis

Endometriosis refers to a condition where endometrial tissue grows outside the uterus in the peritoneal cavity and ovaries [37]. It is an estrogen-dependent chronic inflammatory disorder that is caused by retrograde menstruation, as well as metaplasia of cells that abnormally differentiate into endometrial cells at ectopic sites with enhanced proliferation and migration hypothetically [38, 39]. Its gold standard diagnosis of endometriosis depends on diagnostic laparoscopy with excisional biopsy and scoring system [40].

Endometriosis appears to affect 5% of the population, peaking at 10% in women at reproductive ages, translating to 176 million women worldwide, with associated symptoms including dysmenorrhea, pelvic pain, dyspareunia and infertility [41, 42]. Statistically, about 5% of infertile women suffer from endometriosis, and 25 to 40% of women with endometriosis are diagnosed as infertile [43]. For patients with infertility, ART serves as one of the most efficient managements of endometriosis and can be performed without previous surgery [44]. However, the impairment of IVF success in women with advanced endometriosis has been reported [45]. A meta-analysis published in 2019 showed classified results that milder forms of endometriosis were most likely to affect the fertilization (FR OR 0.77, CI 0.63–0.93) and earlier implantation processes (implantation rate OR 0.76, CI 0.62–0.93), whilst stage III-IV endometriosis significantly decreased live births in women undergoing IVF (with an odds ratio of 0.78, 95% CI 0.65–0.95) [46]. Considering systemic effects especially metabolic disorders caused by endometriosis, researchers turned to metabolomics to obtain metabolic profiles as fingerprints for perturbation characteristics of endometriosis and furthermore, to explore biomarkers to improve the IVF outcome of patients with endometriosis [47, 48].

Analytical methods such as tandem mass spectrometry and sequential window acquisition of all theoretical fragment-ion spectra visualized the differences in the metabolomic profiles between endometriosis patients and their counterparts with other kinds of infertility. Sphingolipids and phosphatidylcholines were found in high abundance in FF from patients with endometriosis, and ChoGpl, the main lipid subclass, was often overexpressed in endometriotic lesions as a substrate for phospholipase A2 (PA2) enzyme [49]. Besides, an upregulated level of LysoPC (18:2(9Z,12Z)) and LysoPC (18:0) along with a downregulated level of phytosphingosine was uncovered in human follicular fluid in patients with endometriosis reported by a case-control study [50]. Lactate, β-glucose, pyruvate and valine were also found to be significantly more abundant in the FF of the women with ovarian endometriosis than those of control group, statistically [51]. In order to understand the association between metabolic alteration and ART outcomes such as oocyte quality, embryo viability and pregnancy rates, further analyses were conducted. A study based on NMR approach evidenced higher levels of phospholipids, lactate, insulin, PTX3, CXCL8, CXCL10, CCL11 and VEGF companied by lower concentrate of some fatty acids, lysine, choline, glucose, aspartate, alanine, leucine, valine, proline, phosphocholine, total LDH as well its LDH-3 isoform in endometriosis group constituting of women who suffered from different stages of endometriosis (I-II and III-IV) and underwent IVF cycles. LDHB, PTX3 and insulin receptor were furtherly confirmed by RT-PCR applied on cumulus cells surrounding oocytes, correlating reduced oocyte quality observed in these patients with endometriosis to the different levels of these metabolic molecules [52]. Metabolic profiles of infertile women with endometriosis were found to be statistically different from fertile women with the same disease, especially in phosphatidic acids (PA) (35:6) and PA (37:7). PA (35:6) refers to an intermediate of the PI molecule, a potential marker for cases of lymphoblastic leukemia while PA (37:7) is considered as an intermediate for the marker for ovarian cancer. PA was found to trigger biochemical pathways that impaired pregnancy outcomes by affecting directly immune system functioning, hormonal environment of the eggs, implantation, and egg quality. By estimating PA concentrate in infertile and fertile woman, the specific metabolite was reinforced as an infertility-related biomarker for endometriosis [53]. Whereas, larger-scale studies and randomized controlled trials are in desperate need to illustrate the pathological mechanism of endometriosis and to assist searching biomarkers for ART outcomes.

## FF Metabolomics in Diminished Ovarian Reserve

The phenomenon that elder women undergoing IVF acquire less numbers of retrieved oocytes and available embryos than younger women may attribute to decrease in ovarian function [54]. Ovarian aging, especially diminished ovarian reserve (DOR), has been acknowledged as one of the most common causes of infertility and represents a major challenge in reproductive medicine. Clinically, infertile women with DOR can be identified by low levels of anti-Müllerian hormone (AMH) below 0.5–1.1 ng/ml, low antral follicle counts (AFC) blow 5–7 and elevated basal follicle-stimulating hormone (FSH) levels higher than 10 IU/L [55].

The prevalence of DOR varied between 10% and 26% among women of reproductive age, with an increasing incidence observed within ART population [56, 57]. The causes of DOR include autoimmune diseases, inherited chromosome and genetic disorders, environmental hazards, iatrogenic causes while a large part of it still remains unexplained [58–60]. Patients with DOR tend to suffer from reproductive decline with exceedingly high rates of pregnancy loss and significantly low rates of clinical pregnancy and live birth in ART therapy [56, 61]. Therefore, biomarkers of DOR seem to be in an urgent need to estimate female reproductive capacity and furthermore, to predict ART outcomes at diverse levels. Genetically, telomere or methylome changes in leukocytes rather than follicular somatic cells (granulosa and cumulus) was verified to correlate with reproductive function and was considered as surrogate biomarkers of women with DOR [62]. At transcriptome level, circulating miR-22-3p in blood distinguished patients with declined ovarian reserve from control counterparts with area under the ROC curve (AUC) 0.668, 95% confidence interval (CI) 0.602–0.733 and its sensitivity and specificity were 75.4% and 54.6% respectively at the cutoff value of 0.607. Additionally, miRNA-21 in FF exhibited high sensitivity of 74.8% and specificity of 83.7% with the AUC value of 0.774, 95%CI 0.682–0.865 (*p* = 0.01) for predicting clinical pregnancy outcomes [63, 64]. At protein level, the concentration of 55 cytokines was reported to be significantly different from DOR to the control group and 44 of these cytokines could be biomarkers of DOR with an AUC of 0.78 [65]. Urinary vitamin D-binding protein (VDBP), the key protein for the protein-protein interaction network, has been identified to be correlated with ovarian reserve and was considered as a novel noninvasive diagnostic biomarker considering its sensitivity of 67.4% and a specificity of 91.8% for DOR patients [66]. Although prior findings showed associations of the ovarian reserve with urinary concentrations of some individual phenols and phthalate metabolites, such as bisphenol A, butylparaben, methylparaben, propylparaben and di(2-ethylhexyl) phthalate, researchers failed to confirm association of these chemicals as a mixture with the ovarian reserve [67]. Due to the fact that targeted research could rarely provide biological information in a large quantity or reflect the complicated reaction as a whole, recently, researchers turned to the metabolomics to search for biomarkers of DOR. Meanwhile, FF rose up as a relatively ideal subject owing to its close relation to oocyte and easiness in obtain.

It is acknowledged that DOR patients have different FF metabolomic profiles from women with normal ovarian reserve. The FF concentrations of pregnanediol-3-glucuronide and 2-hydroxyestrone sulfate which were primarily enriched in the choline pathway from DOR group were testified to be significantly different from FF of normal ovarian reserve (NOR) group [68]. Besides, lower glucose, ascorbate and GSH values as well as higher lactate, hypoxanthine, xanthine, uracil, cytosine, and cytidine, MDA, 8-OH-dG, nitrite and nitrate values were also found in FF of DOR in comparison to control group (q < 0.005), based on Biomarker Score values developed by Lazzarino, G. etc. [69]. With AUC value of 0.9952, top ten out of twenty metabolites involved in aminoacyl-tRNA biosynthesis, tryptophan metabolism, pantothenate and CoA biosynthesis, and purine metabolism were successfully integrated to build a diagnostic model to predict ovarian function [22]. More recently, 15 oxylipins metabolites were also found to be lower in the FF of DOR patients than those in control group. Among them, ± 20-HDoHE (AUC = 0.782), 12 S-HHTrE (AUC = 0.762), 20-COOH-AA (AUC = 0.758), 8 S,15 S-DiHETE (AUC = 0.800), PGA2 (AUC = 0.850), and PGE1 (AUC = 0.818) in FF showed high sensitivity and specificities for ovarian reserve function. In order to explore biomarkers of IVF outcomes for DOR patients, 9 different oxidized lipid metabolites (20-HDoHE, ± 5-iso PGF2α-VI, 12 S-HHTrE, 8 S,15 S-DiHETE,1a,1b-dihomo PGE2, 20-COOH-AA, PGA2, PGE1 and PGF2α) were furtherly analyzed and testified to be positively correlated with DOR markers such as AMH, the number of oocytes retrieved, MII oocytes and fertilization, whilst only 20-COOH-AA was positively associated with the number of high-quality embryos after ART [12].

Metabonomic study on the FF of patients with DOR not only facilitates DOR diagnosis with prediction model but also provides data support for the research of the pathogenesis of DOR as well as promising biomarkers for predicting IVF outcomes.

## FF Metabolomics in Uterine Factors

Uterine cavity abnormalities which affect 20–50% of women of childbearing potential, are associated with poor pregnancy outcomes including decreased chances of achieving live birth and increased risks of miscarriage in clinically diagnosed women [70, 71]. Common abnormalities in uterine cavity include endometrial polyps, uterine leiomyoma, intrauterine adhesions, and congenital uterine malformations. Surgery such as polypectomy, curettage and hysteroscopic metroplasty are currently administered as a routine to improve reproductive outcomes of infertile women with uterine cavity abnormalities such as endometrial polyp and cavity-distorting defects [72, 73].

For the last decade, metabolomic study on uterine cavity abnormalities has facilitated clinical practice with considerate diagnostic and therapeutic information. Among 14 metabolites screened out by gas chromatography coupled to mass spectrometer, amino acids (L-isoleucine, L-valine, and pyroglutamic acid), fatty acids (arachidonic acid, alfa-tocopherol, palmitic acid, and stearic acid) and carbohydrates (myo-inositol, D-threitol, and D-ribose) were found to be reduced in plasma of patients with large leiomyomas whilst L-glutamine and alpha-linolenic acid were significantly increased, conferring a better understanding of leiomyoma’s pathophysiology [74]. Further study filtrated four specific biomarkers, 6-keto-PGF1α, PA (37:4), LysoPC (20:1) and PS (36:0) that showed preferable classification and diagnostic ability in diagnosis of endometrial polyp with AUC of 0.915, sensitivity of 100% and specificity of 72.41% [75]. In regard to clinical treatment of infertility, metabolomics also unveiled the abnormal improvement of 51 differential biomarkers in intrauterine adhesion rats and discovered significant improvement after prunella vulgaris oil treatment, illustrating the pharmacological results in alleviating inflammation and fibrosis on intrauterine adhesion models [76]. On purpose of predicting IVF outcomes, metabolomics was also utilized to seek for valuable bio-information in FF, a by-product of IVF process that could serve as an economic, non-invasive object for metabolomic research. In FF samples collected from women who had uterine problems and achieved pregnancy after IVF procedures, the contents of six differential metabolites were screened out to be statistically different from those of non-pregnant women, including GlcCerC (d18:0/20:0), phosphatidylethanolamine, PC (16:0/16:0), PG (O-18:0/16:1(9Z)), PE (O-18:0/22:2(13Z,16Z)) and GluCer d18:1/24:0. Furthermore, these analytes were acknowledged to be involved in cell signaling, growth regulation and were also defined as potential markers for cancers [53]. Apart from this, in subfertile patients with endometrial polyps, decreased creatine and increased lactate signals were demonstrated as evidence of impaired receptivity [77].

These findings by metabolomics allow a deeper and thorough understanding of the pathophysiological processes of uterine abnormality and provide insights for the development of personalized approaches to improve implantation outcomes. Nevertheless, more reliable biological markers to predict embryo implantation and pregnancy afterwards are needed to improve IVF outcomes.

## FF Metabolomics in Tubal Factors

Tubal dysfunction is ranked as the leading reason for female infertility, accounting for 30–35% of female acyesis causes [78]. Tubal infertility refers to the inability to conceive caused by fallopian impairment in both structure and function, usually owing to acute and chronic pelvic infection, pelvic and abdominal surgery, postoperative adhesions, tubal tuberculosis and endometriosis [79, 80]. As a result of pathological alteration of fallopian tubes, including swelling, thickening, adhesion, stiffness, occlusion and hydrosalpinx, the gametes and zygote delivery is interrupted. For the evaluation of tubal patency, conventional hysterosalpingography (X-HSG), MR-hysterosalpingography (MR-HSG) and three-dimensional hysterosalpingo-foam sonography (3D-HyFoSy) are well developed and widely used to guide further treatment such as laparoscopic surgery and IVF [81, 82].

Tubal infertility is an exclusive and unmixed pathology type to investigate diagnosis, pathophysiology and therapy of infertility. It keeps bringing substantial progress to this field. By analyzing FF samples of patients with tubal factors, the changes of FF lipid composition were found to be altered with age and their relation to the quality of oocytes were discovered [83]. In search of IVF predictors, study on tubal factors showed positive correlation between tryptophan and the available embryo rate and lysoPE (16:0/0:0) along with DG (18:2(9Z,12Z)/15:0/0:0) was screened out to indicate pregnancy rate, delivery rate and live birth rate [36, 84]. It was also demonstrated that FF has different metabolic characteristics in different stages of follicular development. Moreover, by studying infertile patients with tubal factors, dehydroepiandrosterone (DHEA) has been examined to estimate follicular development and was claimed as a potential predictor correlating with rates of oocyte maturation and high-quality embryo [85]. Noticing endocrine influence on metabolomics, our previous metabolomic study on infertile patients specially excluded PCOS, endometriosis and other metabolism-related diseases and successfully screened out resolvin E1 as a potential biomarker to preclude inferior oocytes by FF resolvin E1 level below 8.96 pg/ml (AUC:0.75; 95%CI: 0.64–0.86; *P* = 0.00012) with specificity of 97.22% [86]. In general, metabolomics exploration in tubal infertility makes great contribution to ART study.

In comparative infertility study, tubal factors are commonly regarded as controls on the hypothesis that tubal abnormality rarely affects endocrinology or ovulation. In order to screen out biomarkers in FF to robustly predict the abortion rate of the PCOS group, infertile patients with fallopian tubal issues only were set as control and consequently 11 kinds of metabolites including androsterone sulfate, glycerophosphocholine, and elaidic carnitine were successfully discovered as predictors [36]. Besides, the finding that sphingolipids and phosphatidylcholines were in relatively high abundance in endometriosis and endometrioma was also depended on comparison with tubal infertility as a control [49].

Besides the method of setting tubal factors as control group, a considerate amount of study established control group with a mixture of both tubal factors and male factors. For example, a study conducted in FF from patients with tubal factor female infertility and/or mild male factor infertility illustrated differential metabolomics representation in FF between pregnant and non-pregnant patients, showing increase of phosphatidic acid, phosphatidylglycerol and triacylglycerol and decrease of glucosylceramide in the pregnant group [87]. It was once claimed impossible to discriminate metabolic profiles between FF of control participants and women with tubal diseases [33]. However, a recent study discovered that infertile women with tubal factor had different metabolic profiles from fertile women and the statistic separation confirmed that the discriminative metabolites were associated with specific infertility problems including PCOS, endometriosis, tubal dysfunction and other factors [53]. More specifically, the discrepancy in FF lipids constitution was found between infertile women with tubal factors and their counterparts with male factors. Additionally, for metabolites like cholesterol, citrate, creatine, β-hydroxybutyrate, glycerol, lipids, amino acids (Glu, Gln, His, Val, Lys) and glucose, significant differences were also detected in FFs of women with male factor while no significant difference was observed in women with tubal diseases. Furthermore, in tubal disease, the number of MII oocytes correlated positively with lipid while in male infertility, it was positively associated with citrate and negatively with glucose [88]. Therefore, in metabolomic study, tubal factors should neither be mixed by other factors such as male factors and unexplained factors as controls nor be equated with healthy controls.

In a word, the metabolomic approach motivates the exploration in infertility and meanwhile provides potential biomarker discovery of IVF. Nevertheless, it needs more rigorous and reasonable criteria to recruit objects and establish control group.

## Discussion

Conventionally, a majority of reproductive centers transfer several embryos in one period to obtain an impressive clinical pregnancy rate, which is regarded as the most convenient and efficient approach. However, transferring more than one embryo at the same time inevitably leads to a high multiple pregnancy rate and consequently results in adverse birth outcomes [89]. The elective single-embryo transfer (eSET) which is currently encouraged, especially the single-blastocyst transfer, helps to decrease the rate of multiple gestations [90]. Meanwhile, the elevated profits of multiple embryo transfer in clinical pregnancy rates fades away when adopting eSET [91]. Despite the mainstream utilization of morphology assessment and PGT before embryo transfer to select qualified embryos to enhance the clinical success of IVF-ET procedures, neither of the pregnancy nor the live birth rates satisfies the patients’ expectation [92]. Therefore, metabolomic study in search of biomarkers for ART outcomes has gained an increasing attention, prospectively laying the foundation for judicious evaluation of IVF outcomes.

Metabolites refers to the low-molecular-weight end products of metabolic reactions that are essential for the function and development of cells. The non-targeted identification and quantification of the complete collection of metabolites in an organism is so-called metabolomics. Comparing to analytical methods such as genomics, proteomics and biopsy, metabolomics as a non-invasive approach, has the advantage of relatively limited kinds of metabolites, minimal sample volume, short analyzing time and thus is able to provide dynamic and comprehensive information [93]. During the last decades, metabolomics has been applied to examine the metabolic profiles of biofluids such as follicular fluid, culture medium and blastocoele fluid, providing clinicians with valuable information on oocyte quality, embryo competence, endometrial receptivity and changes on fertility due to cancer, assisting understanding and appraising the micro-environment where gametes developed in ART for infertile patients with common infertility-related diseases and reproductive failure such as early miscarriage, recurrent miscarriage, and repeated implantation failure [94–97]. From simple ANNOVA, partial least squares-discriminant analysis models, metabolomics scoring methods to cutting-edge deep-learning and even AI, metabolomics keeps developing and innovating, facilitating the prediction of IVF outcomes with novel perspectives and constantly updated results [98–100]. Although implementing metabolomic techniques in daily clinical routines still faces a series of practicle difficulties, including high costs and the demand for specialized professional staff, it is worthy of further exploration and development.

However, it was once doubted that metabolomic study of biofluid or tissues possesses no potential to improve fertility outcomes and consequently their use in clinical practice has been limited [101]. It might be intelligible when considering the great heterogeneity in study conducted in different centers all over the world. First of all, the species distinction between animals and humans needs to be considered when drawing conclusions. Secondly, inconsistent variable standardization of methods and varied technical levels among respective clinical centers also add to the incredibility. Besides, limited sample sizes of the underlying datasets and lack of validation in external populations may account for the potential factors that affects study quality [102]. Last but not the least, current research is devoid of categorical statements according to specific causes of infertility populations. In view of the fact that different basic infertile diseases have unique metabolic profiles and may lead to differed metabolomic alteration which affects the metabolic discrimination between superior and inferior quality oocytes, it seems illogical and inaccurate to estimate the predicting efficacy of metabolomics in indicating IVF outcomes without classification of infertility causes. Therefore, in this review, we classified and analyzed existing research to assess the role of metabolomic investigation in improving clinical pregnant outcomes in women undergoing according to their differed pathologic categories.

FF, the biofluids that fills the antral cavity surrounding the oocyte in follicles, serving as the in vivo microenvironment for oocytes, contains all of the essential substances and nutrition for follicle growth and oocyte maturation [103]. Moreover, FF can be easily obtained since it is aspirated at the time of oocyte pick-up and is supposed to be discarded. Its performance of being objective, easy-obtained, non-invasive and causing minimal influence on IVF itself render FF an ideal object for assessment. Ovarian FF was firstly analyzed with employment of NMR in 1990 [104]. Since then, FF has played an unsubstituted role in reflecting oocyte quality. Although study on FF in exploration of IVF biomarkers has been reported in infertile women with PCOS, endometriosis, DOR, tubal malfunction, and even uterine abnormality, there still lacks metabolomic analysis on FF collected from patients suffering from other kinds of subfertility causes such as cervical abnormality, male factors and unexplained factors. It tends to be easy to correlate metabolism disorder with typical kinds of infertility that are caused by endocrine disorder or has the manifestation of endocrinopathy and consequently it seems reasonable to turn to metabolomics to further current research. Tubal factors, which has been regarded to have no relation with metabolic disorder and has been used as control in various ART research, was discovered to have differed metabolic profiles from normal counterparts [88]. Indeed, in subgroup of tubal infertility, the FF metabolomics differed from women who achieved pregnancy after IVF procedures to those who failed to conceive [87]. Similarly, it might be reckless to exclude ‘non-metabolic’ infertility such as uterine and cervical abnormality, male factors and unexplained factors when conducting metabolomic study on FF from IVF patients. When utilizing FF as study object, for accuracy of research, potential for FF contamination should be noticed [17]. In ovary collection, FF sample might be contaminated by flushing medium and blood that contains numerous metabolites and thus the real metabolic constitution of the FF would be disturbed. Besides, FF may also be contaminated by previously aspirated FF, which casts a considerate challenge to FF biomarkers research. Therefore, FF sample should be aspirated from the specific follicle to explore clinical outcomes of the specific oocyte and embryo and more importantly, only uncontaminated FF sample should be adopted.

In this review, by investigating the current status of metabolomics study in ART and pointing out the potential drawbacks in its research and application, we propose the construction of an aggregation panel to collect all biomarkers discovered through metabolomic estimation. Furthermore, variables should be standardized, trials be properly conducted, and uploaded statistics need to be correctly classified according to biological species and specific infertile subgroups with different etiologies. Only on the basis of panel like this, may conventional and neoteric data processing gain robust computing power, inventing an objective and effective tool for outcome prediction and meanwhile, justify the clinical value of metabolomics.

## Data Availability

All data and paper reviewed in this study could be found in web.
